# Silencing long intergenic non-protein coding RNA 00987 inhibits proliferation, migration, and invasion of osteosarcoma cells by sponging miR-376a-5p to regulate FNBP1 expression

**DOI:** 10.1007/s12672-021-00412-x

**Published:** 2021-06-01

**Authors:** Riliang Cao, Jianli Shao, Wencai Zhang, Yongxin Lin, Zerong Huang, Zhizhong Li

**Affiliations:** Department of Orthopedics, First Affiliated Hospital of Jinan University, 613 W. Huangpu Avenue, Tianhe District, Guangzhou, 510630 China

**Keywords:** FNBP1, lncRNA, miRNA, Osteosarcoma

## Abstract

High expression of long intergenic non-protein coding RNA 987 (*LINC00987*) is strongly associated with low overall survival of osteosarcoma; however, its role in osteosarcoma remains unclear. This study explored the biological function and underlying mechanism of *LINC00987* in osteosarcoma. In this study, *LINC00987* expression in osteosarcoma cells was analyzed using Cancer Cell Line Encyclopedia and qRT-PCR. The proliferation and migration and invasion in osteosarcoma cells were evaluated using Cell Counting Kit-8 and Transwell assays, respectively. Bioinformatic analysis was used to predict the *LINC00987*-bound miRNAs and miR-376a-5p-bound mRNAs. Dual-luciferase reporter assays were used to assess the interaction between miR-376a-5p, *LINC00987*, and forming-binding protein 1 (FNBP1). FNBP1 expression was measured by western blotting. *LINC00987* was found to be upregulated in osteosarcoma cells. *LINC00987* silencing suppressed proliferation, migration, and invasion of osteosarcoma cells. Additionally, miR-376a-5p expression was downregulated in osteosarcoma cells. miR-376a-5p knockdown reversed the effect of *LINC00987* silencing on the biological function of osteosarcoma cell. miR-376a-5p was found to target *LINC00987* and FNBP1. FNBP1protein level was increased in osteosarcoma cells; however, it was inhibited by silencing *LINC00987* and enhanced by silencing miR-376a-5p. In conclusions, this study suggests *LINC00987* silencing inhibits osteosarcoma cell proliferation, migration, and invasion by sponging miR-376a-5p to regulate FNBP1 expression. *LINC00987* as a potential therapeutic target for osteosarcoma.

**Supplementary Information:**

The online version contains supplementary material available at 10.1007/s12672-021-00412-x.

## Introduction

Osteosarcoma is a common malignant bone tumor with lung metastasis, high mortality, and poor prognosis and mainly occurs in children and adolescents under 20 years of age [1, 2]. To date, surgery combined with neoadjuvant chemotherapy has been the major treatment for osteosarcoma [3]. However, the surgical plan is established; therefore, drug-assisted treatment is the key to improving the survival rate. Hence, further study of the complex pathogenesis of osteosarcoma will help develop novel drugs.

Long noncoding RNAs (lncRNAs) can regulate tumorigenesis, metastasis, and gene expression in various cancer tissues or cells [4, 5]. Abnormal expression of lncRNAs involved in tumorigenesis, progression, metastasis, and chemotherapy drug resistance can be used as therapeutic targets and diagnostic and prognostic markers in osteosarcoma [6–8]. A previous study found that high long intergenic non-protein coding RNA 987 (*LINC00987*) expression strongly correlated with low survival of osteosarcoma [9]. However, their biological functions in osteosarcoma remain unclear.

In this study, the expression of *LINC00987* in osteosarcoma cells was analyzed. Next, we focused on further investigating the role and regulatory mechanisms of *LINC00987* to provide new references for treating osteosarcoma.

## Materials and methods

### Cell culture

The conditionally immortalized human fetal osteoblastic cell line hFOB1.19 (normal cell) and human osteosarcoma cell lines (143B, U2OS, MG63, and Saos-2) were purchased from the Institute of Cell Bank/Institutes for Biological Sciences (Shanghai, China). Cells were cultured as described in our previous study [10].

### qRT-PCR assay

Total RNA was extracted from hFOB1.19, 143B, MG63, Saos-2, and U2OS cells using TRIzol reagent (Invitrogen, Carlsbad, CA, USA) and dissolved in 20 μL DEPC H_2_O. The first-strand cDNA synthesis reaction system was configured in an RNase-free PCR tube, and mRNA was reverse-transcribed to cDNA using the EasyScript First-Strand cDNA Synthesis SuperMix kit (Transgen, Beijing, China). lncRNA, miRNA, and mRNA expression was detected using SYBR Green qPCR SuperMix (Invitrogen) with the ABI PRISM 7500 Sequence Detection System (Applied Biosystems, Foster City, CA, USA). β-actin was used as an endogenous control for lncRNA and mRNA, while *U6* was used as a miRNA. All experiments were performed in triplicate. The relative expression was calculated using the 2^−ΔΔCt^ method [11]. PCR primer sequences were purchased from Sangon (Shanghai, China) and are listed in Table 1. Each experiment was repeated three times.

**Table 1:** Synthesized sequences

*LINC00987*	siRNA sequence	
	Sense (5′–3′)	Antisense (5′–3′)
si-*LINC00987*-1	GCUCUGUGCAUCAGGUAAATT	UUUACCUGAUGCACAGAGCTT
si-*LINC00987*-2	GGAUUUAGCCUUGUGCCAATT	UUGGCACAAGGCUAAAUCCTT
si-*LINC00987*-3	GGUAAUGAAUGGUGACUAUTT	AUAGUCACCAUUCAUUACCTT

qRT-PCR	Primer sequences	
	Forward primer (5′–3′)	Reverse primer (5′–3′)
*LINC00987*	GTTCTGATTTCCTGGGTTACC	TTCCTAGAACTCACCCTTCCT
*FNBP1*	ATTGGGAAGTGCCTGGATGG	TCTGACACAGTGCGCTTCAT
β-actin	GCATGGGTCAGAAGGATTCCT	TCGTCCCAGTTGGTGACGAT
miR-370-3p	ACACTCCAGCTGGGGCCTGCTGGGGTGGAACC	CTCAACTGGTGTCGTGGA
miR-376a-5p	ACACTCCAGCTGGGGTAGATTCTCCTTCTATGA	CTCAACTGGTGTCGTGGA
miR-543	ACACTCCAGCTGGGAAACATTCGCGGTGCACT	CTCAACTGGTGTCGTGGA
*U6*	CTCGCTTCGGCAGCACA	AACGCTTCACGAATTTGCGT

### Transfection

Three *LINC00987* siRNAs (si-*LINC00987*-1, -2, and -3) and a negative control siRNA (si-NC) were purchased from Shanghai Genepharma (Shanghai, China) and are shown in Table 1 (Shanghai, China). Thereafter, 50 ng each of si-NC, si-*LINC00987*-1, si-*LINC00987*-2, and si-*LINC00987*-3 was transfected into 143B and U2OS cells, respectively. After transfection for 48 h, *LINC00987* expression was measured by qRT-PCR. Moreover, miR-376a-5p inhibitor (miR inhibitor) and negative control inhibitor (NC inhibitor) were purchased from Shanghai GenePharma (Shanghai, China). Thereafter, 50 ng each of si-*LINC00987*-3 and NC inhibitor (si-LINC00987-3 + NC inhibitor) or miR inhibitor (si-*LINC00987*-3 + miR inhibitor) was co-transfected into 143B or U2OS cells, respectively. After transfection for 48 h, miR-376a-5p expression was measured by qRT-PCR.

### Cell proliferation, migration, and invasion assays

For cell proliferation assay, 24 h after transfection, MG-63 and Saos2 cells were seeded at 1 × 10^3^ cells per well in 96-well plates. The cell proliferation assay was performed at 24, 48, and 72 h using the Cell Counting Kit-8 (CCK8; Dojindo, Kumamoto, Japan) at 450 nm. Transwell migration and invasion assays were performed as previously described [10]. The bottom of the upper chamber was pre-coated with or without Matrigel (BD Biosciences, San Diego, CA, USA) for migration and invasion assays. The transfected cells (5 × 10^4^ cells) in 200 µL of 0.1% fetal bovine serum (FBS)-containing medium were placed in the upper chamber. The lower chamber was filled with 10% FBS (600 µL). After 48 h of culture, cells in the upper chamber were removed. The cells that passed through the membrane filter were fixed and stained and then randomly observed (200 × magnification) using a LEICA Microscope (Tokyo, Japan) for each well.

### Bioinformatic database assay

The Cancer Cell Line Encyclopedia (CCLE) database (www.portals.broadinstitute.org/ccle) was used to analyze the levels of *LINC00987* transcripts in osteosarcoma cells. The miRNA expression in osteosarcoma cells was analyzed by using GEO2R in GSE70367 of Gene Expression Omnibus (GEO, https://www.ncbi.nlm.nih.gov/geo/), and the downregulated expression of miRNAs (fold change ≤ −1) was selected. The potential miRNAs bound to *LINC00987* were analyzed using the LncBase Experimental v.2 subset of DIANA Tools [12]. The potential mRNAs bound to miR-376a-5p were analyzed using Targetscan 7.2 [13], miRDB [14], and miRWalk (http://mirwalk.umm.uni-heidelberg.de/), and the intersection was taken for further verification. Additionally, the intersection mRNA expression in osteosarcoma cells was analyzed using GEO2R in GSE70367 and Oncomine (https://www.oncomine.org/).

### Plasmid construction and luciferase reporter assay

The 3′-untranslated region (UTR) sequences of wild-type *LINC00987* and forming-binding protein 1 (*FNBP1*) (WT-*LINC00987* and WT-*FNBP1*) and mutated *LINC00987* and *FNBP1* (MUT-*LINC00987* and MUT-*FNBP1*) were synthesized by GENEWIZ (Suzhou, China) and cloned into the luciferase reporter vector psi-CHECK2. For the luciferase reporter assay, 293 T cells were plated at a density of 5 × 10^4^ cells per well in 24-well plates. The next day, 293 T cells were co-transfected using Lipofectamine 2000 (Invitrogen) with the following combinations: WT-*LINC00987* plus NC mimic or miR-376a-5p mimics, MUT-*LINC00987* plus NC mimic or miR-376a-5p mimics, WT-*FNBP1* plus NC mimic or miR-376a-5p mimics, and MUT-*FNBP1* plus NC mimic or miR-376a-5p mimics. Forty-eight hours after transfection, luciferase assays were performed using a dual-luciferase reporter assay system (Promega).

### Western blotting

Western blotting was performed as described previously [10]. Briefly, total proteins were extracted from osteosarcoma cells. Extracted proteins (30 µg) were separated using 10% SDS-PAGE and transferred onto a polyvinylidene difluoride membrane (Millipore, Billerica, MA, USA). After blocking, membranes were incubated with anti-FNBP1 antibody (Cat# ab100918, 0.5 µg/mL, Abcam, Cambridge, MA, USA) for 1 h at 37 °C. After washing in TBS with 0.5% Tween 20, the membrane was incubated with a horseradish peroxidase-conjugated secondary antibody. Finally, chemiluminescence detection was performed using enhanced chemiluminescence, and signals were recorded on X-ray films.

### Statistical analysis

Statistical analysis was performed using the SPSS software (version 19.0; IBM, Chicago, IL, USA). Normally distributed data are expressed as the mean ± standard deviation. The differences between multiple groups (more than two groups) were compared using one-way ANOVA followed by Tukey’s post-hoc test. The differences between the two groups were compared using *t-*tests. Statistical significance was set at P < 0.05.

## Results

### *LINC00987* expression is increased in osteosarcoma cells

**Fig. 1:**
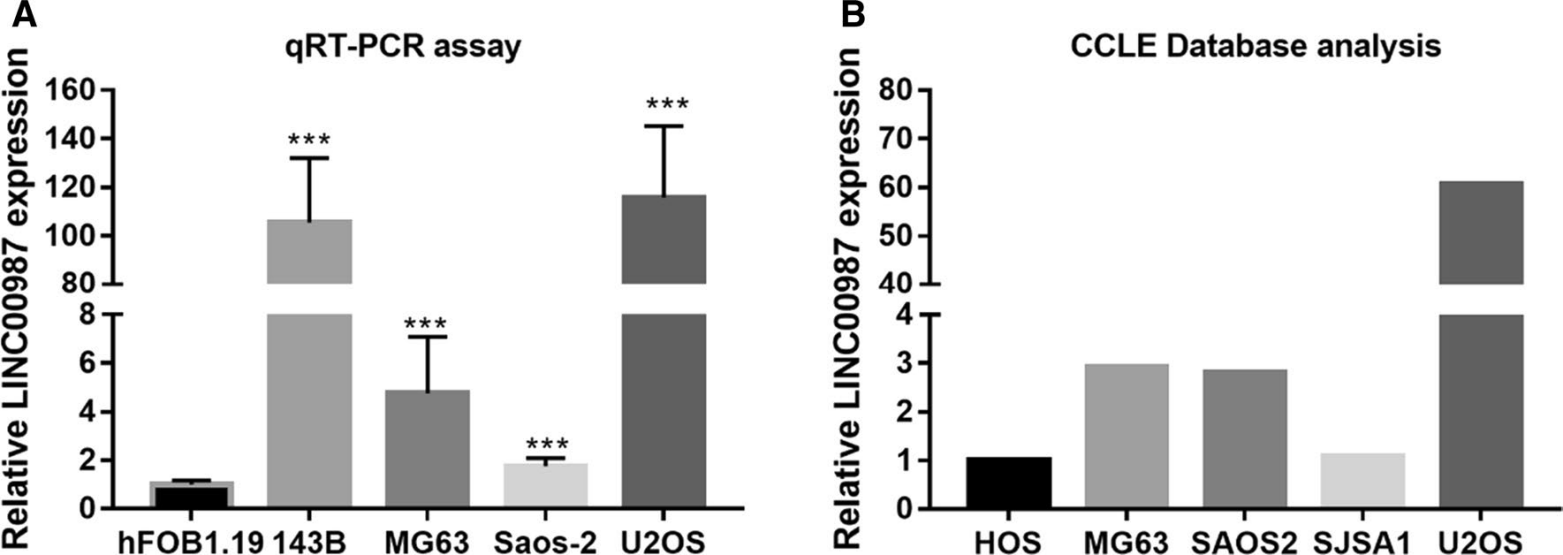
*LINC00987* expression is increased in osteosarcoma cells, especially in 143B and U2OS cells. **A** LINC00987 expression in the conditionally immortalized human fetal osteoblastic cell line hFOB1.19 (normal cell) and human osteosarcoma cell lines (143B, U2OS, MG63, and Saos-2) measured using qRT-PCR. ***P < 0.001 vs. hFOB1.19 group. **B** *LINC00987* expression in osteosarcoma cell lines were analyzed using the Cancer Cell Line Encyclopedia (CCLE) Database

The qRT-PCR results showed that *LINC00987* expression in osteosarcoma cells (143B, U2OS, MG63, and Saos-2) was significantly higher than that in hFOB1.19 cells (normal cell) (Fig. 1A). A previous study found that higher *LINC00987* expression was strongly correlated with the lower overall survival of osteosarcoma [9]. In this study, the expression of LINC00987 was consistent with previous studies. Additionally, *LINC00987* expression in 143B and U2OS cells was significantly higher than that in in MG63 and Saos-2 cells (Fig. 1A). The results from CCLE-database analysis showed that *LINC00987* expression in U2OS cells was obviously higher than that in other osteosarcoma cells (Fig. 1B). Hence, this study selected 143B and U2OS cells for further investigations.

### *LINC00987* silencing inhibits proliferation, migration, and invasion of 143B and U2OS cells

**Fig. 2:**
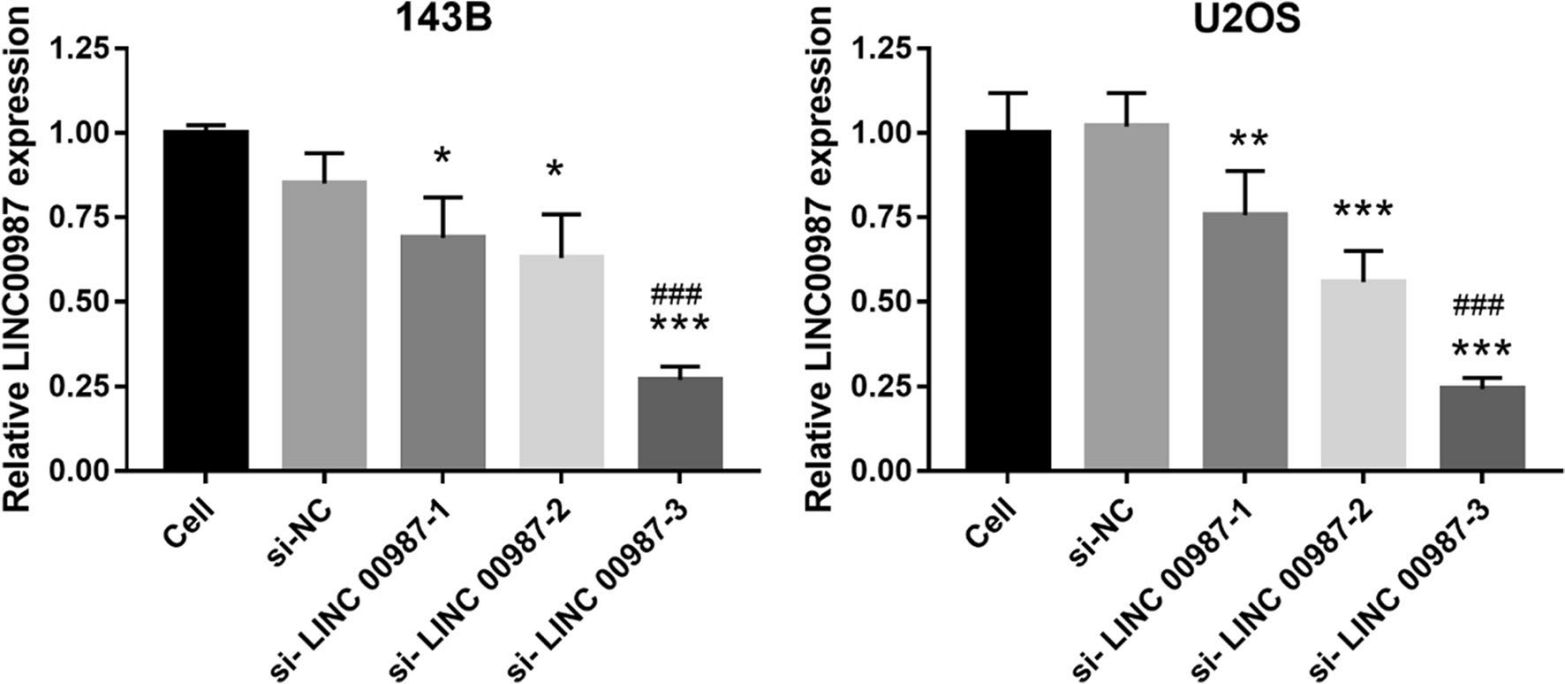
*LINC00987* expression is inhibited by *LINC00987* siRNA (si-*LINC00987*) transfection. *LINC00987* expression was measured by qRT-PCR 24 h after transfection of 143B and U2OS cells with three si-*LINC00987* sequences and negative control siRNA (si-NC). ***P < 0.001 vs. si-NC group

**Fig. 3:**
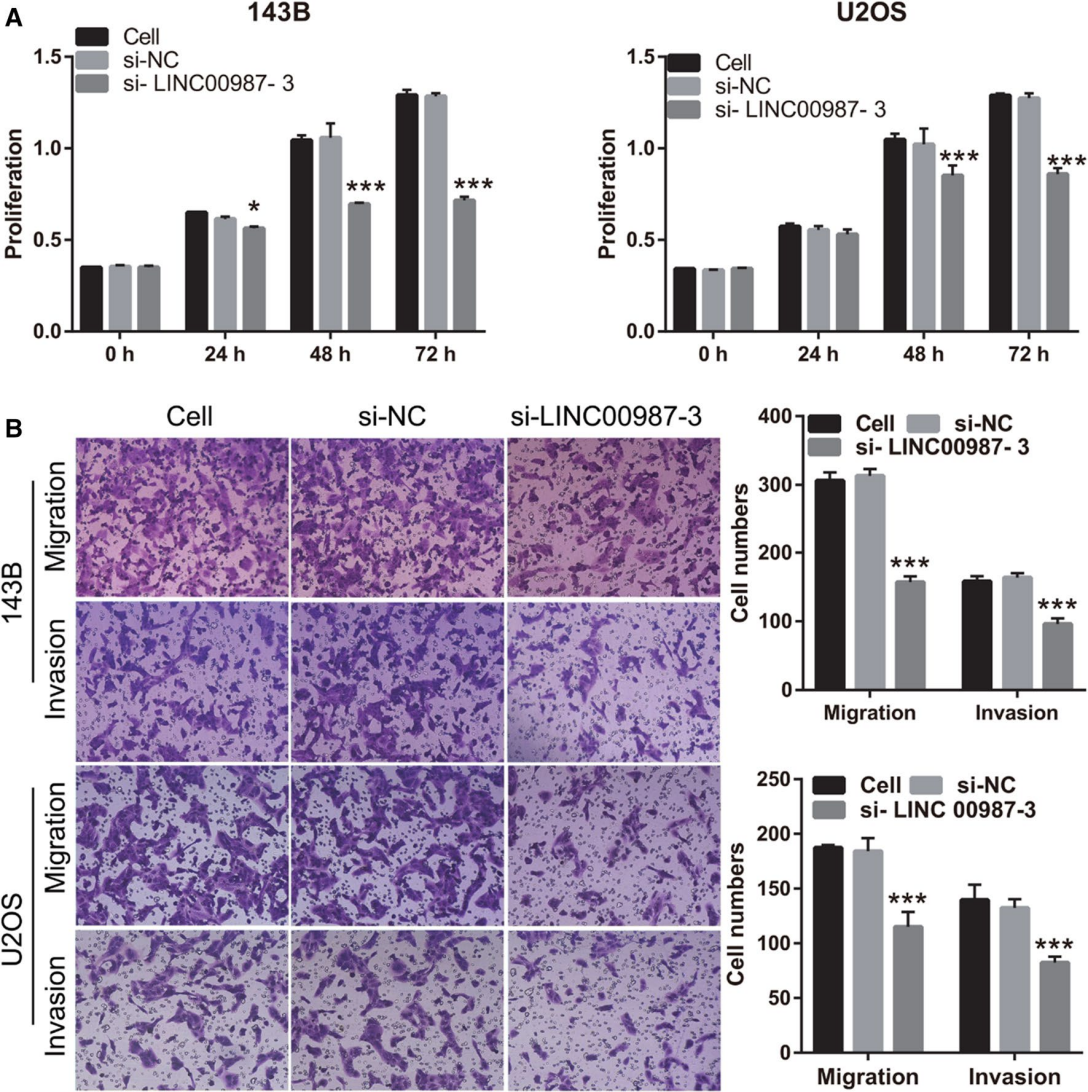
Silencing of *LINC00987* inhibits the proliferation, migration, and invasion of 143B and U2OS cells. **A** Proliferation measured 24, 48, and 72 h after transfection, using CCK8. **B** Migration and invasion measured using Transwell assay 48 h after transfection. *P < 0.05, ***P < 0.001 vs. si-NC group

To study the effect of *LINC00987* on the biological function of osteosarcoma cells, *LINC00987* expression was knocked down in 143B and U2OS cells. Compared with the cell group, si-NC group showed no significant change in *LINC00987* expression, whereas the si-*LINC00987* group showed significantly reduced *LINC00987* expression (Fig. 2). Compared with the si-*LINC00987-1* group, the si-*LINC00987*-2 group showed no significant change in *LINC00987* expression, while the si-*LINC00987*-3 group showed significantly reduced *LINC00987* expression (Fig. 2). Hence, si-*LINC00987*-3 transfection was selected for further analysis. Additionally, compared with the cell group, the si-NC group showed no significant change in proliferation, migration, and invasion, while the si-*LINC00987* group showed significantly reduced proliferation, migration, and invasion, after transfection at 48 and 72 h (Fig. 3).

### *LINC00987* acts as sponge for miR-376a-5p

**Fig. 4:**
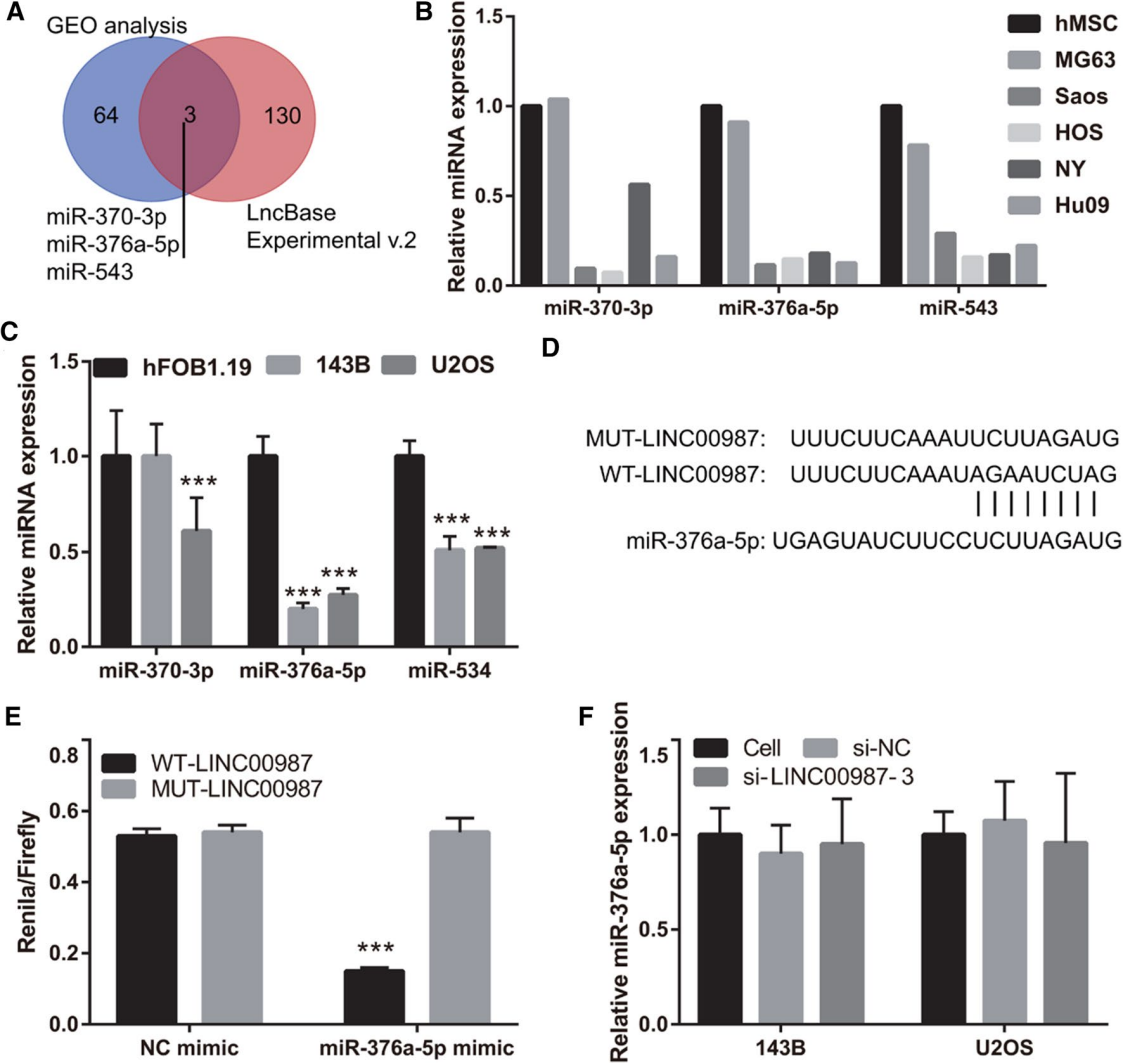
*LINC00987* acts as sponge for miR-376a-5p. **A** The intersection between the potential miRNAs analyzed using LncBase Experimental v.2 and downregulated-expression miRNAs analyzed using GEO. **B** miRNA expression in osteosarcoma cells and human mesenchymal stem cells (hMSCs) analyzed using GEO. **C** miRNA expression in osteosarcoma cells and the conditionally immortalized human fetal osteoblastic cell line hFOB1.19 measured using qRT-PCR. **D** The wild-type and mutated binding sites between miR-376a-5p and *LINC00987*. **E** Luciferase reporter assay results. **F** miR-376a-5p expression in 143B and U2OS cells measured using qRT-PCR 24 h after transfection

The intersection between the potential miRNAs analyzed by LncBase Experimental v.2 and downregulated miRNAs analyzed by GEO are shown in Fig. 4A. The results showed that miR-370-3p, miR-376a-5p, and miR-543 might bind to *LINC00987*. Moreover, miR-370-3p, miR-376a-5p, and miR-543 expression was lower in all osteosarcoma cells, except for MG63 cells, than in human mesenchymal stem cells (hMSC), according to the GEO-analyzed results (Fig. 4B). miR-376a-5p and miR-543 expression was significantly reduced in 143B and U2OS cells, and miR-370-3p expression was significantly reduced in U2OS cells, whereas there was no significant change in 143B cells, compared to those in hFOB1.19 cells (Fig. 4C). In addition, the fold reduction in miR-376a-5p expression was lower than that of miR-543 (Fig. 4C). Hence, we chose miR-376a-5p for further studies. The wild-type and mutated binding sites between miR-376a-5p and *LINC00987* are shown in Fig. 4D. Co-transfection of miR-376a-5p mimic inhibited the relative luciferase activity of WT-*LINC00707*-transfected cells, whereas it did not affect the activity of MUT-LINC00707-transfected cells (Fig. 4E). Furthermore, miR-376a-5p expression in 143B and U2OS cells did not differ between the cell, si-NC, and si-*LINC00987*-3 groups (Fig. 4F). These results showed that miR-376a-5p expression was reduced in 143B and U2OS cells and that miR-376a-5p can bind to *LINC00987*.

### miR-376a-5p reverses the effect of *LINC00987* in 143B and U2OS cells

**Fig. 5:**
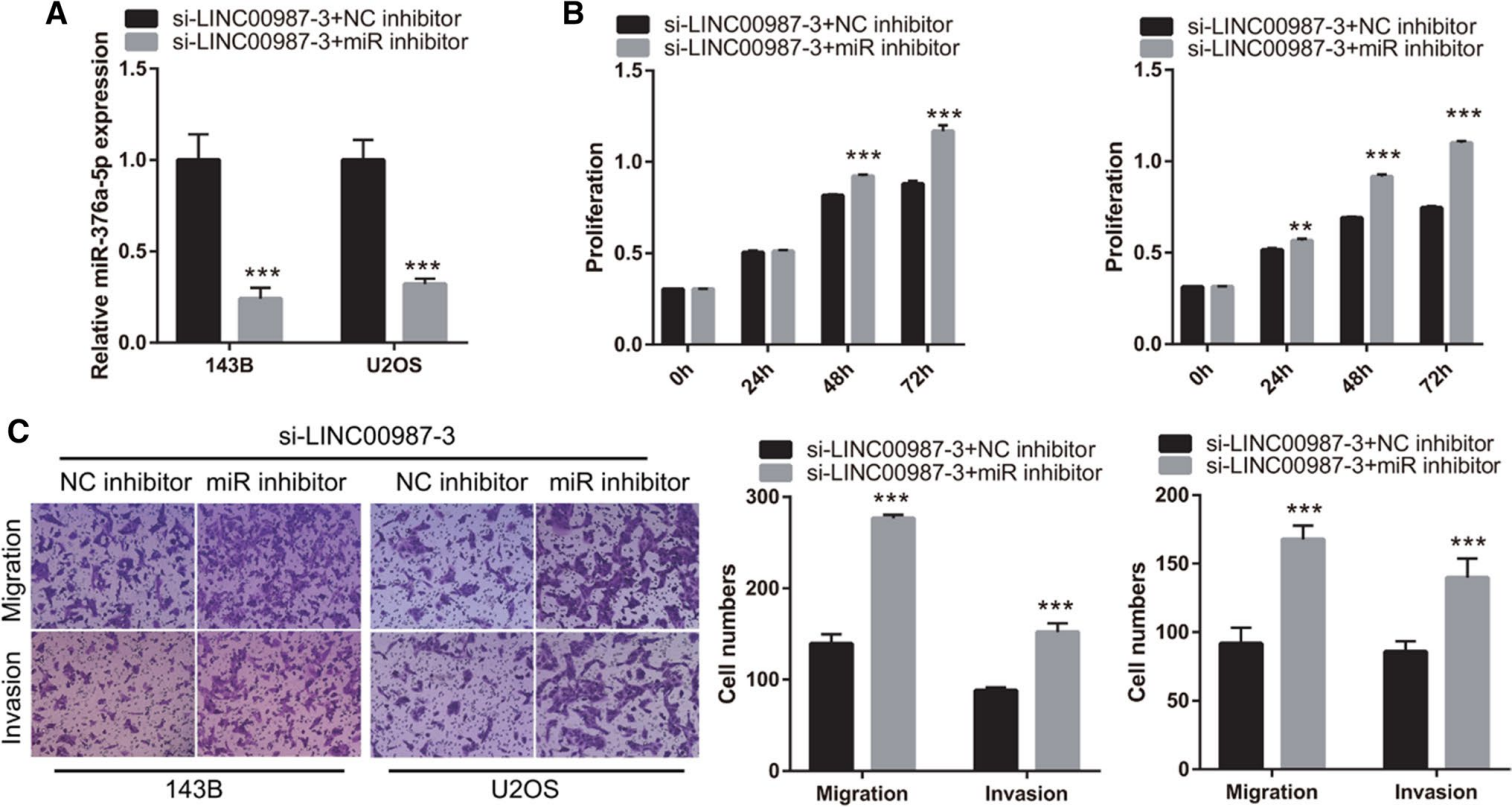
miR-376a-5p reverses the effect of *LINC00987* in 143B and U2OS cells. si-*LINC00987*-3 and NC inhibitor (si-*LINC00987*-3 + NC inhibitor) or miR-376a-5p inhibitor (si-*LINC00987*-3 + miR inhibitor) were co-transfected in 143B and U2OS cells, respectively. **A** miR-376a-5p expression was measured using qRT-PCR 24 h after transfection. **B** Proliferation measured 24, 48, and 72 h after transfection, using CCK8. **C** Migration and invasion measured using Transwell assay 48 h after transfection. **P < 0.01, ***P < 0.001, vs si-LINC00987-3 + NC inhibitor

To study the relationship between *LINC00987* and miR-376a-5p, si-*LINC00987*-3 + NC inhibitor and si-*LINC00987*-3 + miR inhibitor were co-transfected in 143B and U2OS cells, respectively. The results of qRT-PCR showed that miR-376a-5pexpression was knocked down in si-*LINC00987*-3-transfected 143B and U2OS cells (Fig. 5A). Cell proliferation was significantly increased in the si-LINC00987-3 + miR inhibitor group after transfection at 48 and 72 h compared to that in the si-*LINC00987*-3 + NC inhibitor group (Fig. 5B). Furthermore, migration and invasion of the si-*LINC00987*-3 + miR inhibitor group were significantly increased compared to those in the si-LINC00987-3 + NC inhibitor group 48 h after transfection (Fig. 5C).

### *FNBP1* is the target of miR-376a-5p

**Fig. 6:**
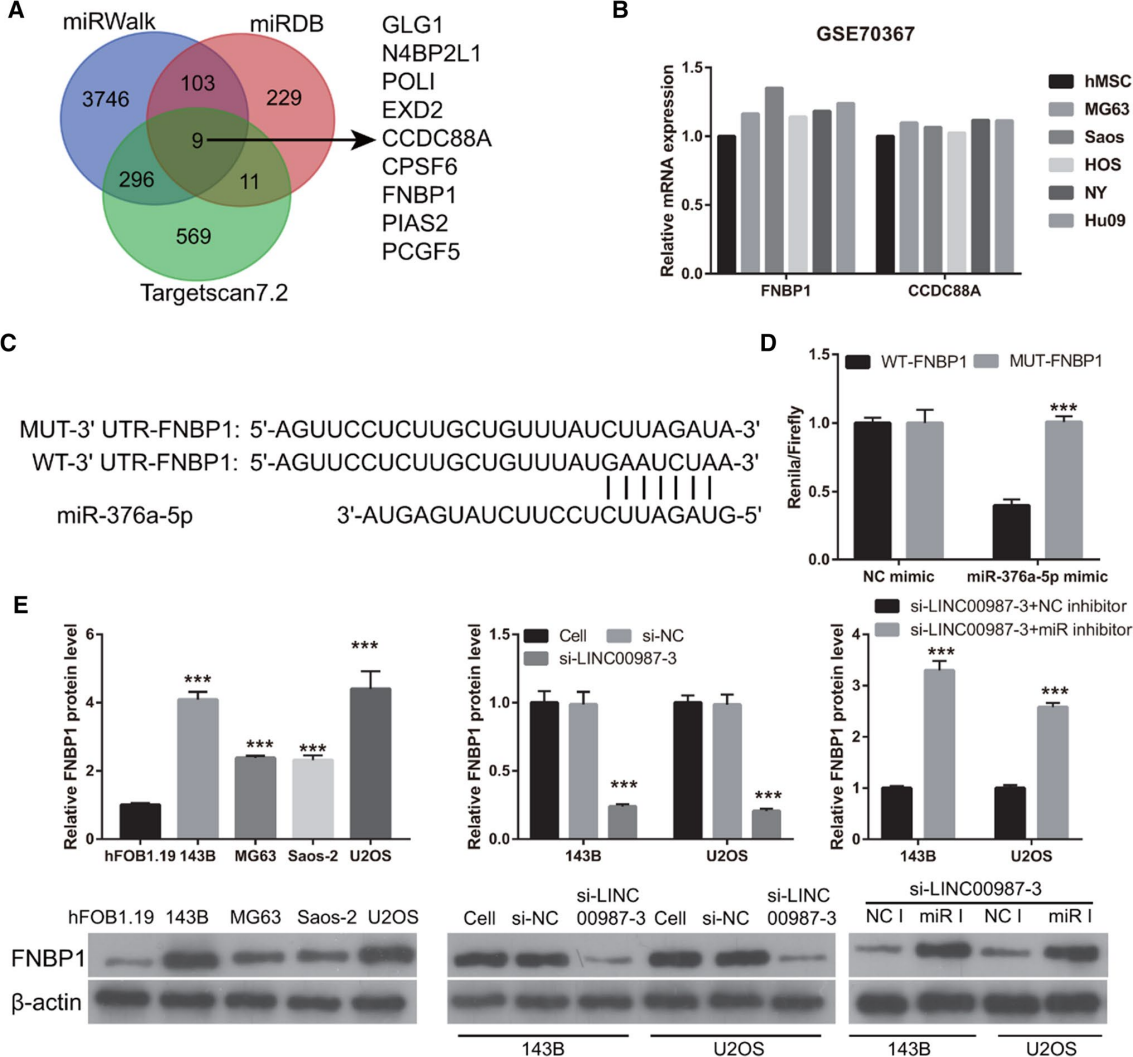
FNBP1 is the target of miR-376a-5p. **A** The intersection between the potential mRNAs analyzed using Targetscan 7.2, miRDB, and miRWalk. **B** *FNBP1* and *CCDC88A* expression in osteosarcoma cells and human mesenchymal stem cells (hMSCs) analyzed using the GEO database. **C** The wild-type and mutated binding sites between miR-376a-5p and FNBP1. **D** Luciferase reporter assay results. **E** mRNA expression and protein level of FNBP1 measured using qRT-PCR and western blotting, respectively, in the conditionally immortalized human fetal osteoblastic cell line hFOB1.19 and human osteosarcoma cell lines (143B, U2OS, MG63, and Saos-2), in 143B and U2OS cells transfected with three si-*LINC00987* sequences and negative control siRNA (si-NC), and in 143B and U2OS cells co-transfected with si-*LINC00987*-3 and NC inhibitor (si-*LINC00987*-3 + NC inhibitor) or miR-376a-5p inhibitor (si-*LINC00987*-3 + miR inhibitor)

The intersection between the potential mRNAs analyzed by Targetscan 7.2, miRDB, and miRWalk is shown in Fig. 6A. The results showed that *FNBP1* and *CCDC88A* mRNA expression was higher in osteosarcoma cells than that in human mesenchymal stem cells, according to the GEO-analyzed results (Fig. 6B). The wild-type and mutated binding sites between miR-376a-5p and FNBP1 3′-UTR are shown in Fig. 6C. Co-transfection of miR-376a-5p mimic inhibited the relative luciferase activity of WT-*FNBP1* 3′-UTR-transfected cells; however, it did not affect MUT-FNBP1 3′-UTR-transfected cells (Fig. 6D). Moreover, the wild-type binding sites between miR-376a-5p and *CCDC88A* 3′-UTR had only six bases, and there was no significant difference in the relative luciferase activity between cells co-transfected with miR-376a-5p mimic and WT-*CCDC88A* 3′-UTR and those co-transfected with miR-376a-5p mimic and MUT-*CCDC88A* 3′-UTR (results not shown). Additionally, compared with normal cell (hFOB1.19 cell), CCDC88A protein levels had no significant change in 143B and MG63 while had significant change in U2OS and Saos-2 (Additional file 1: Fig. S1). These results indicated that miR-376a-5p cannot target CCDC88A 3'-UTR and the expression trends of CCDC88A and LINC00987 in osteosarcoma are not similar. Hence, CCDC88A is not a target gene regulated by *LINC00987*/miR-376a-5p. FNBP1 protein levels were significantly increased in osteosarcoma cells, particularly in 143B and U2OS cells, compared to those in hFOB1.19 cells, which was similar with the expression trends of *LINC00987* (Fig. 6E). Compared with the cell group, the si-LINC00987-3 group showed significantly increased FNBP1 protein levels (Fig. 6E). And compared with the si-LINC00987-3 + NC inhibitor group, the si-LINC00987-3 + miR-376a-5p inhibitor group showed significantly decreased FNBP1 protein levels (Fig. 6E). These results suggest that FNBP1 is the target of miR-376a-5p. And *LINC00987* and miR-376a-5p can regulate FNBP1 protein levels.

## Discussion

**Fig. 7:**
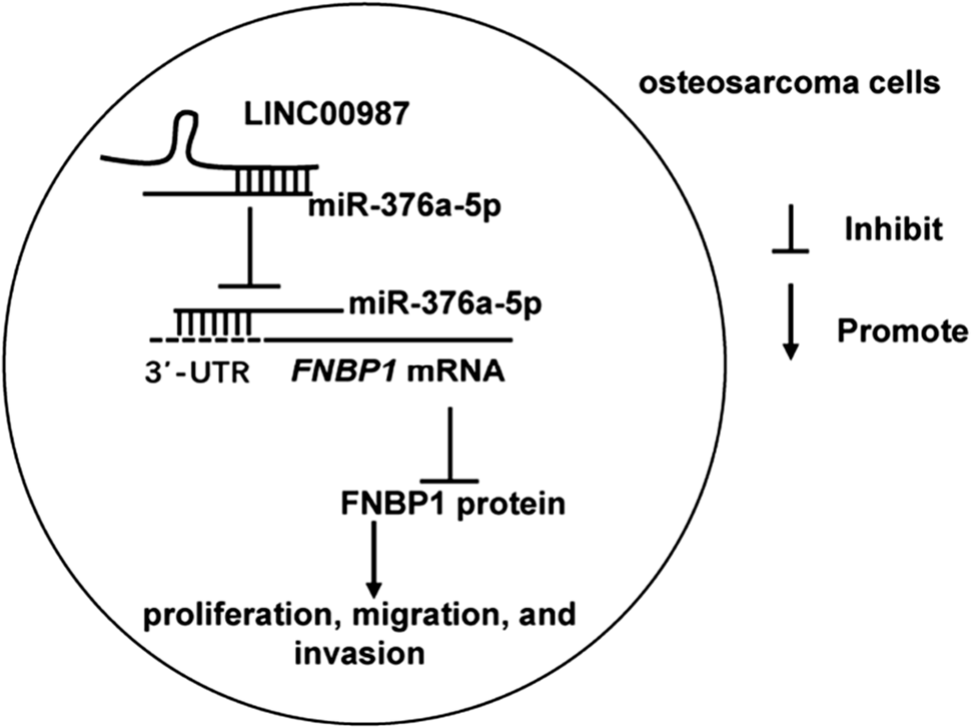
Mechanism diagram: LINC00987 reduces the binding of miR-376a-5p and 3′-UTR FNBP1 by sponging miR-376a-5p, thereby increasing the level of FNBP1 protein and promoting the proliferation, migration and invasion of osteosarcoma

The remission and treatment of osteosarcoma have significantly improved over the last 10 years [2]. However, osteosarcoma does not have a favorable prognosis because of unresolved problems, such as complex pathogenesis and lack of new adjuvant drugs. Therefore, further studies on the mechanism of osteosarcoma development are necessary to develop new therapeutic targets and adjuvant therapeutic drugs. This study focused on the mechanistic involvement of *LINC00987* in osteosarcoma. First, it was found that *LINC00987* silencing inhibited the proliferation, migration, and invasion of 143B and U2OS cells. Moreover, the inhibition of miR-376a-5p remarkably recovered cell proliferation, migration, and invasion in *LINC00987*-silenced cells. Additionally, this study found that FNBP1 is the target of miR-376a-5p. *FNBP1* expression was increased in osteosarcoma cells; however, it was inhibited by silencing *LINC00987* and enhanced by silencing miR-376a-5p. The results revealed an abundance of *LINC00987* through sponging miR-376a-5p and positive regulation of FNBP1 protein level, consequently contributing to the proliferation, migration, and invasion of osteosarcoma cells (Fig. 7).

Osteosarcoma overgrowth and metastasis are the most important biological characteristics of osteosarcoma [15]. However, the potential mechanisms underlying these phenomena have not been completely elucidated. The effect of abnormal lncRNA expression in osteosarcoma has received increasing attention in fundamental studies [16]. This study found that *LINC00987* expression is enhanced in osteosarcoma cells and that inhibition of *LINC00987* expression inhibited the proliferation, migration, and invasion of osteosarcoma cells. This study provides evidence that *LINC00987* is an oncogene and therapeutic target. A previous study found that *LINC00987* expression was increased in patients with acute myeloid leukemia and osteosarcoma and was closely related to poor prognosis [9, 17]. These studies also support that *LINC00987* is an oncogene, similar to our results.

lncRNAs act as competitive endogenous RNAs to sponge miRNAs and regulate their target mRNA expression transcriptionally or posttranscriptionally in osteosarcoma [18, 19]. In this study, we found that *LINC00987* acts as a sponge for miR-376a-5p. Previous study found that miR-376a-5p expression was reduced in lymphoma and miR-376a-5p knockdown enhanced lymphoma proliferation and apoptosis by targeting FOXP2 [20]. Additionally, miR-376a-5p reduced glioblastoma migration and invasion [21]. There result showed that miR-376a-5p is an antioncogene in lymphoma and glioblastoma. However, effect of miR-376a-5p in osteosarcoma has not been elucidated. This study was the first to report that miR-376a-5p expression was reduced in osteosarcoma cells and that silencing miR-376a-5p promotes osteosarcoma development and attenuates the anticancer effect of *LINC00987* inhibition in osteosarcoma. These results showed that miR-376a-5p is an antioncogene in osteosarcoma. Furthermore, it was found that miR-376a-5p targets FNBP1 (also known as FNBP17), a member of the forming-binding protein family. *FNBP1* expression was increased in pediatric medulloblastoma, which may act as prognostic markers and therapeutic targets [22]. Previous studies found that *FNBP1* was overexpressed in invasive breast cancer cells and ductal carcinomas, regulating the cytoskeleton and metastasis [23, 24]. *FNBP1* promoted invadopodia formation and invasive capacity in bladder tumor cells [25]. These results showed that *FNBP1* expression is increased in pediatric medulloblastoma, breast cancer cells and ductal carcinomas, and bladder tumor, which is an oncogene. Similarly, in this study, we found that *FNBP1* is overexpressed in osteosarcoma. Additionally, silencing of *LINC00987* inhibited FNBP1 levels, whereas silencing miR-376a-5p promoted FNBP1 levels, through posttranscriptional regulation in osteosarcoma. These results highlight that *LINC00987* regulates the proliferation, migration, and invasion of osteosarcoma cells by adsorbing miR-376a-5p and posttranscriptionally regulating the expression of FNBP1.

## Conclusion

Silencing *LINC00987* inhibits proliferation, migration, and invasion of osteosarcoma cells by sponging miR-376a-5p to regulate FNBP1 expression. This study provides a potential therapeutic target for osteosarcoma.

## Supplementary Information

**Additional file 1: Figure S1.** CCDC88A protein levels had significant overexpression in Saos-2 and U2OS. CCDC88A protein level was measured by western blot. * P < 0.001 vs. hFOB1.19 cell (normal cell).

## Data Availability

All data generated or analysed during this study are included in this published article [and its Additional file].

## Abbreviations

*LINC00987*: Long intergenic non-protein coding RNA 987
*987FNBP1*: Forming-binding protein 1
lncRNA: Long noncoding RNA
NC: Negative control
FBS: Fetal bovine serum
CCLE: Cancer Cell Line Encyclopedia
GEO: Gene Expression Omnibus
3′- UTR: 3′-Untranslated region
WT: Wild-type
