# Supplementary material for: Silencing long intergenic non-protein coding RNA 00987 inhibits proliferation, migration, and invasion of osteosarcoma cells by sponging miR-376a-5p to regulate FNBP1 expression
Source: Discov Oncol. 2021 Jun 1;12:18. doi: 10.1007/s12672-021-00412-x (PMC8777572; doi:10.1007/s12672-021-00412-x)
Supplement: Supplementary file 1 — Additional file 1: Figure S1. CCDC88A protein levels had significant overexpression in Saos-2 and U2OS. CCDC88A protein level was measured by western blot. * P < 0.001 vs. hFOB1.19 cell (normal cell). [file 12672_2021_412_MOESM1_ESM.docx]

Additional file 1: Figure S1. FNBP1 protein levels had significant overpression in MG63, and Saos-2. FNBP1 protein level was measured by western blot. * P < 0.001 vs. hFOB1.19 cell (normal cell).
